# From Daily Attitudes to Alcohol‐Related Harm: A Within‐Person Serial Mediation Test Informed by the Theory of Planned Behavior

**DOI:** 10.1111/acer.70431

**Published:** 2026-10-27

**Authors:** Angelo M. DiBello, Jennifer E. Merrill, Kate B. Carey, Mary Beth Miller

**Affiliations:** ^1^ Center of Alcohol and Substance Use Studies Graduate School of Applied and Professional Psychology Rutgers University New Brunswick New Jersey USA; ^2^ Center for Alcohol and Addiction Studies Brown University Providence Rhode Island USA; ^3^ Department of Psychiatry University of Missouri Columbia Missouri USA; ^4^ Department of Psychological Sciences University of Missouri Columbia Missouri USA

**Keywords:** alcohol, alcohol‐induced amnesia, alcohol‐related consequences, blackout, mediation

## Abstract

**Background:**

The Theory of Planned Behavior (TPB) posits that attitudes influence behavior through intentions, yet this pathway has not been thoroughly examined at the within‐person level in the alcohol literature. This study tested whether daily fluctuations in attitudes toward heavy drinking predicted same‐day alcohol‐related consequences and alcohol‐induced blackouts through a serial mediation involving daily drinking intentions and alcohol consumption.

**Methods:**

Participants were 87 heavy‐drinking young adults (53.4% female; *M*age = 22.22 years) enrolled in the intervention arm of a randomized clinical trial who completed up to 30 consecutive days of morning diary assessments. Multilevel models separated within‐and between‐person variance by person‐mean centering daily predictors. Serial mediation pathways were estimated using multilevel regression models, and specific indirect effects were tested using Monte Carlo confidence intervals.

**Results:**

On days when participants reported more favorable attitudes toward heavy drinking than their own average, they also reported stronger intentions to drink, which predicted greater same‐day alcohol consumption. Greater drinking, in turn, predicted increased odds of both alcohol‐related consequences and blackout. Significant serial indirect effects supported the hypothesized pathway from attitudes to consequences and blackout through intentions and drinking. Drinking intentions also directly predicted alcohol‐related consequences independent of drinking quantity but did not directly predict blackout after accounting for drinking, suggesting differences in the mechanisms underlying these outcomes.

**Conclusion:**

Findings provide day‐level support for a TPB‐informed pathway linking within‐person fluctuations in drinking attitudes to alcohol‐related harm through drinking intentions and alcohol consumption. Results extend prior between‐person TPB research by demonstrating that attitudes and intentions function as dynamic, day‐to‐day processes that help explain when individuals are at elevated risk for harmful drinking outcomes. Daily attitudes and intentions may represent promising targets for ecological momentary interventions designed to reduce alcohol‐related harm.

## Graphical abstract

This daily dairy study examined the covariation of daily shifts in favorable attitudes toward heavy drinking, drinking intentions, alcohol use, and alcohol‐related harm among young adults who drink heavily. On days when participants held more favorable attitudes toward heavy drinking than their own average, they reported stronger intentions to drink, more alcohol consumption and, in turn, higher odds of alcohol‐related consequences and blackout. These findings highlight daily attitudes and intentions as proximal and potentially modifiable targets for reducing alcohol‐related harm.

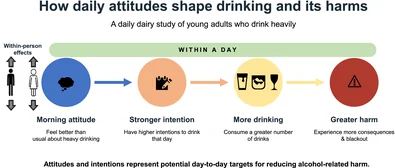

## 1 Introduction

Heavy alcohol use among young adults remains a significant public health concern, as it is associated with a range of negative outcomes including alcohol‐induced blackouts (Wetherill and Fromme 2016). Approximately half of young adults who use alcohol report having experienced at least one blackout, defined as a partial or complete memory loss resulting from heavy alcohol consumption, and these instances of memory impairment are associated with severe harms, including injury and emergency room visits (Hingson et al. 2016; Mundt et al. 2012; Wetherill and Fromme 2016). Understanding the cognitive and motivational mechanisms that translate into harmful drinking behavior is critical for developing effective prevention and intervention strategies. The Theory of Planned Behavior (TPB; Ajzen 1985) offers a well‐validated framework for identifying such mechanisms, positing that attitudes, social norms, and perceived behavioral control predict intentions, which in turn drive behavior.

### 1.1 Attitudes Toward Drinking as a Strong Predictor of Alcohol‐Related Outcomes

Within the TPB framework, attitudes are said to be global evaluative judgments of a behavior, reflecting the degree to which an individual appraises the behavior as positive or negative. Attitudes are distinct from alcohol‐related expectancies, which capture specific anticipated outcomes of drinking; rather, attitudes represent a summary evaluation that integrates cognitive and affective appraisals and can influence behavior both directly and indirectly through intention formation (Ajzen 1991; Albarracin et al. 2014). A meta‐analysis by Cooke et al. (2016) found strong support for TPB constructs, especially attitudes, in predicting intentions, which in turn predict heavy episodic drinking as well as drinking quantity. Growing research has documented attitudes toward heavy drinking as a particularly robust and consistent predictor of alcohol use and its consequences, demonstrating associations with drinks per week, alcohol‐related consequences, and heavy episodic drinking, both cross‐sectionally and prospectively (Collins and Carey 2007; DiBello et al. 2022; Elliott and Ainsworth 2012; Huchting et al. 2008). Notably, the predictive utility of attitudes toward heavy drinking persists even when accounting for other well‐established predictors (e.g., drinking motives, drinker identity) and other theoretically relevant constructs such as descriptive norms and drinking intentions (DiBello et al. 2022, 2018).

Comparatively less work has extended TPB to more severe, acute outcomes of heavy drinking, such as alcohol‐induced blackouts. Blackouts are an important outcome to isolate, as they pose significant risk for other alcohol‐related harms between people (Hingson et al. 2016), over time (Studer et al. 2019), and within‐persons at the day‐level (Merrill et al. 2019). However, to date, only one study has applied the full TPB model to the study of blackout. DiBello et al. (2020) found that blackout‐specific attitudes, norms, and self‐efficacy predicted intentions to experience a blackout, which in turn predicted blackout frequency both concurrently and one month later, with attitudes emerging as the sole TPB variable exerting a significant prospective indirect effect on blackout frequency through intentions, controlling for level of drinking. Notably, however, that work assessed cognitions at the blackout‐specific level (e.g., attitudes toward blacking out). Because blackouts and other acute consequences are downstream effects of the same behavior, heavy drinking, general attitudes toward heavy drinking may represent a more proximal and ecologically common antecedent. Indeed, general attitudes likely capture favorable evaluations of heavy drinking that increase consumption and, in turn, contribute to alcohol‐related harm that is often unintended. These findings position attitudes as a particularly potent antecedent in the chain leading to alcohol‐related harm. However, the within‐person pathways remain understudied. Specifically, it is unknown whether individuals are more likely to experience blackout or other alcohol‐related harms on *days* when their attitudes toward drinking are more favorable, or their intentions to drink are stronger, relative to their typical levels.

### 1.2 Within‐Person Variability in Attitudes

The between‐ versus within‐person distinction is important because the TPB has been tested predominantly at the between‐person level, despite proposing cognitive mechanisms that may also operate within individuals over time. Despite accumulating evidence for a prominent role of attitudes in predicting alcohol use, most research has treated attitudes as static between‐person characteristics, overlooking the possibility that attitudes fluctuate within individuals over time. Attitudes are not necessarily fixed; daily experiences and situational cues may shape attitudes at the momentary level, which in turn may influence same‐day drinking behavior. This has been acknowledged in accessibility models, which suggest that attitudes vary in their activation depending on situational factors and cognitive resources (Fazio 1990, 2007). In addition, constructive perspectives emphasize that evaluative judgments are assembled from currently salient considerations, goals, and affective inputs (Wilson and Hodges 2013). From this perspective, within‐person fluctuations in attitudes toward heavy drinking may reflect context‐dependent shifts in the perceived desirability of drinking rather than wholesale changes in stable evaluative structure.

Initial evidence for within‐person variability in attitudes has emerged from two recent studies. In one study examining attitudes and norms over 12 months (DiBello et al. 2025), mandated college students reported more drinking, greater odds of blackout, and more consequences during periods when they also reported more favorable attitudes toward heavy drinking than their own average. In contrast, favorable attitudes toward moderate drinking were protective (i.e., linked to less drinking and fewer consequences). A subsequent investigation extended this work to the daily level, using a 30‐day diary design among heavy‐drinking young adults (DiBello et al. 2026). In that analysis, days with more favorable heavy‐drinking attitudes than usual carried greater odds of any drinking, more drinks per drinking day, and greater odds of high‐intensity drinking, consequences, and blackout. Together, these two studies suggest that the TPB predictions regarding the role of attitudes that are observed in between‐subjects studies are also supported in within‐subjects studies.

### 1.3 The Role of Intentions in Linking Attitudes to Drinking

Within the TPB, intentions represent the most proximal cognitive antecedent of behavior, serving as the mechanism through which attitudes translate into action. At the between‐person level, attitudes toward heavy drinking predicted prospective change in blackout frequency mediated by intentions (DiBello et al. 2023), consistent with evidence for the attitude–intention–alcohol behavior chain that has been well‐documented (Cooke et al. 2016). Despite robust evidence for between‐person associations, the within‐person relationship between daily intentions and drinking has received comparatively limited attention. However, studies examining intentions alongside behavioral willingness as day‐level predictors of young adult alcohol use found that within‐person fluctuations in drinking intentions were associated with same‐day alcohol use and alcohol‐related consequences, such that stronger‐than‐usual intentions predicted a greater likelihood of drinking, higher alcohol consumption, and greater risk of consequences (Courtney and Russell 2023; Lewis et al. 2020).

Building on these findings, this study extends prior daily process research by examining whether intentions serve as the mechanism linking daily fluctuations in drinking attitudes to subsequent alcohol use and alcohol‐related harm. Intentions assessed close in time to behavior should be particularly strong predictors of deliberative action because they capture the motivational state immediately preceding the behavioral decision (Sheeran 2002). Understanding whether daily intentions mediate the within‐person attitude–drinking relationship would therefore provide important evidence for the TPB‐informed pathway through which attitudes influence drinking outcomes at the day level.

### 1.4 Drinking as a Mediator of Harmful Outcomes

Another key gap in the existing literature concerns the mechanism through which attitudes and intentions translate into downstream alcohol‐related harm. Alcohol‐related consequences and blackouts are strongly influenced by drinking quantity; that is, individuals generally must consume sufficient alcohol to experience most alcohol‐related harms (Hingson et al. 2016; Wetherill and Fromme 2016). However, existing studies often model drinking quantity as a covariate when predicting consequences and blackouts, which estimates the effects of cognitive antecedents above and beyond consumption but does not directly test the behavioral pathway through which these cognitions contribute to harm. Modeling drinking behavior as an explicit mediator rather than a covariate allows for examination of distinct pathways through which attitudes may influence harm: indirectly through intentions, through increased alcohol consumption, and through the full serial pathway linking attitudes, intentions, drinking, and consequences. This approach also allows for the detection of partial mediation, indicating that cognitive antecedents may influence alcohol‐related harm through pathways not fully captured by the proposed model.

### 1.5 The Present Study

This study builds on and integrates evidence from an emerging body of work by examining a TPB‐informed serial mediation model of drinking behaviors at the daily level. Using data from a clinical trial in which heavy‐drinking young adults completed 30 days of daily diaries (Miller et al. 2025), this secondary analysis tests whether within‐person fluctuations in drinking attitudes predict same‐day alcohol‐related consequences and blackouts through a serial chain of same‐day drinking intentions and same‐day alcohol consumption (see Figure 1). Although this study utilizes the same daily diary dataset as DiBello et al. (2026), it addresses a distinct research question and involves no overlapping primary analyses. Specifically, this study extends prior work in three important ways. First, whereas prior studies examined attitude effects over longer intervals (DiBello et al. 2025) and as direct predictors of daily alcohol outcomes (DiBello et al. 2026), this study examines drinking intentions as the mechanism through which daily attitudes influence drinking behavior. Second, we model same‐day alcohol consumption as an explicit mediator linking attitudes and intentions to alcohol‐related harm, providing a novel extension of the TPB behavioral pathway at the daily level. Third, we examine two distinct indicators of harm, alcohol‐related consequences and blackouts, allowing us to evaluate whether cognitive antecedents operate similarly across different types of alcohol‐related outcomes.

**FIGURE 1:**
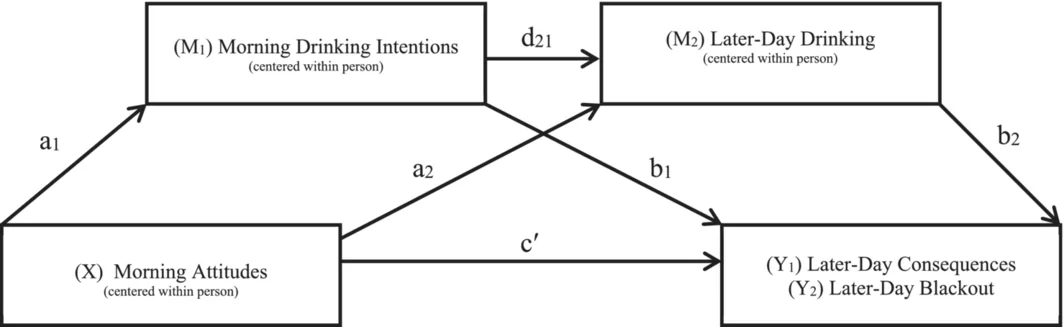
Conceptual serial mediation model.

We hypothesize that (1) on days when attitudes toward drinking are more favorable than a person's own average, daily intentions to drink will be higher; (2) higher daily intentions will predict greater same‐day drinking; (3) greater same‐day drinking will predict a higher likelihood of consequences and blackout; and (4) the serial indirect effect of attitudes on outcomes through intentions and then drinking will be significant.

## 2 Method

### 2.1 Participants and Procedure

Participants were young adults recruited through online digital advertisements for a study on alcohol use and memory between May 2023 and April 2024 (Miller et al. 2025). To be eligible, individuals had to: (a) be between 18 and 30 years of age; (b) reside in the United States; (c) report at least one alcohol‐induced blackout in the prior month; and (d) report either four or more heavy drinking episodes (4+ drinks for women, 5+ drinks for men) or four or more blackouts in the prior month. For this analysis, participants also had to be randomized to the intervention group, as the control group did not complete daily reports on alcohol attitudes or intentions.

Interested individuals completed an online eligibility screen, and those who qualified provided informed consent prior to enrollment. Enrolled participants completed a baseline survey assessing demographic and alcohol‐related variables, after which they were randomized to either a Drinking Dashboard intervention or a screen time control condition. Both conditions completed brief daily morning surveys for 30 consecutive days, delivered at 6:00 a.m. and available through midnight each day. Participants in the control condition completed daily reports focused on screen time only, while participants in the Drinking Dashboard intervention condition completed daily morning alcohol reports. Intervention participants also received personalized weekly feedback summaries of their morning report data. Follow‐up assessments were completed at 30 days and three months post‐baseline. Participants could earn up to $220 in compensation.

Of 777 individuals screened for the parent trial, 290 were excluded due to not meeting the screening criteria, and 239 could not be reached when contacted; this resulted in 248 who completed the baseline assessment. An additional 5 opted out and 74 were excluded prior to randomization for the following reasons: did not complete the verification check‐in (*n* = 9), did not complete diaries (*n* = 13), inconsistent responding (*n* = 4), no past‐month blackout (*n* = 13), no weekly heavy episodic drinking (*n* = 23), repeat participation (*n* = 1), and enrollment exceeding the target sample (*n* = 11). Thus, 169 participants were randomized: 87 to the Drinking Dashboard condition and 82 to the screen time control. As noted above, primary analyses were conducted among the 87 intervention participants.

### 2.2 Measures

#### 2.2.1 Demographics

Participants reported sex assigned at birth (0 = female, 1 = male) and current enrollment in a four‐year college or university (0 = no, 1 = yes).

#### 2.2.2 Daily Attitudes Toward Drinking

Each morning, participants rated their attitude toward heavy drinking using a single item adapted from prior research (DiBello et al. 2022). The item was tailored to present sex‐specific cut‐offs for heavy episodic drinking; specifically, 4+ drinks for females and 5+ drinks for males. Participants responded to the prompt, “Having [4/5] or more drinks in a sitting over the next day would be…” Response options ranged from 1 (bad) to 5 (good).

#### 2.2.3 Daily Drinking Intentions

Each morning, participants reported their intention to drink that day by responding to the item, “How likely are you to drink alcohol today?” Responses were recorded on a 5‐point scale ranging from 0 (*not at all*) to 4 (*almost certainly*).

#### 2.2.4 Daily Alcohol Consumption

Each morning, participants reported the number of standard drinks consumed the previous day. A standard drink reference image was provided to support accurate reporting.

#### 2.2.5 Daily Alcohol‐Related Consequences

Each morning, participants indicated whether they had experienced any of eight alcohol‐related consequences as a result of drinking the previous day (i.e., I embarrassed myself, I threw up, I hurt or injured myself or someone else, I became aggressive, I went to the ER or hospital, I passed out, I had a hangover, something else happened). Due to the relatively low frequency of consequences at the daily level, responses were dichotomized as no consequences (0) versus any consequence (1).

#### 2.2.6 Alcohol‐Induced Blackout

Blackout was assessed separately from general consequences, given its theoretical centrality to the parent study. Participants were trained on the definition of en bloc blackout during study orientation; specifically, being unable to remember events that occurred while drinking, even when prompted by others. Each morning, participants responded to the item, “Did you experience a blackout yesterday?” Responses were dichotomized as no (0) and yes (1).

#### 2.2.7 Descriptive Norms

Perceptions of peer alcohol consumption were assessed at baseline using an adapted version of the Descriptive Norms Rating Form (Baer et al. 1991). Participants estimated the number of standard drinks consumed by a typical person of their same age and sex on each day of a typical week (Monday through Sunday). Responses across the seven days were summed to create a single index reflecting perceived peer drinks per week.

### 2.3 Analytic Strategy

#### 2.3.1 Analyses Overview and Variable Centering

All analyses were conducted in SAS version 9.4 using multilevel modeling frameworks selected to match the distributional properties of each outcome. Because the data were nested (days within persons), each daily Level‐1 predictor was separated into its within‐ and between‐person components. For every daily variable, a within‐person deviation score was created by subtracting each participant's own across‐days mean from their daily values, capturing how much a given day departed from that person's typical level. Consistent with standard within‐person centering (Curran and Bauer 2011), the daily drinking variable was calculated as each day's drink count minus the participant's own average across all diary days. Non‐drinking days were retained and coded as negative deviations from the participant's mean (e.g., a value of −5 for a participant averaging 5 drinks per day), ensuring that all days were represented on a common deviation metric. Each participant's mean was then entered separately as a Level‐2 covariate, removing stable individual differences so that the within‐person coefficient reflects day‐to‐day variation alone.

The focal predictor was daily attitude toward heavy drinking, with the person‐mean retained at Level‐2. The two mediators were daily drinking intentions and daily alcohol consumption, with their person‐means included as Level‐2 covariates. Remaining covariates were sex, study day (1–30), weekend day (0 = non‐weekend; 1 = weekend), and baseline descriptive drinking norms (Level‐2). Descriptive norms were included as a L2 covariate because they are cited as a theoretical determinant of behavior in the TPB; however, we did not assess norms at the daily level.

#### 2.3.2 Multilevel Modeling Analytic Plan

The hypothesized pathway is that within‐person elevations in daily attitude predict same‐day drinking outcomes through their sequential influence on same‐day drinking intentions and, in turn, same‐day alcohol consumption, consistent with a 1‐1‐1‐1 serial mediation structure (Zhang et al. 2009). Because each morning report asked participants to recall the prior day's drinking and its outcomes, the drinks per day, consequences, and blackout variables were lagged by one day (t–1) so that they aligned temporally with the attitudes and intentions reported on the corresponding day. This adjustment ensured that attitudes and intentions temporally preceded their hypothesized outcomes. A three‐step approach was used to partition the total effect of attitude into its component paths (a_1_/a_2_, d_21_, and b_1_/b_2_; see Figure 1). Because two alcohol‐related harm outcomes were of interest (blackout and other consequences), the b_1_/b_2_ paths were estimated separately for each, producing two complete serial models. All models specified a random intercept for participant to accommodate the dependence among repeated daily observations. Random slopes for Level‐1 predictors were tested but the associated models did not converge, indicating too little between‐person variability in slopes to estimate them reliably; we therefore specified random intercepts only across all models.

##### 2.3.2.1 Step 1 Path a_1_: Daily Attitude Predicting Same‐Day Intentions

Daily drinking intentions were regressed on within‐person centered attitude using PROC GLIMMIX with a Gaussian distribution and all covariates noted above. Skew (0.75) and kurtosis (−0.20) values for within‐person centered drinking intentions suggested an approximate normal distribution (Curran et al. 1996; |skewness| < 2, |kurtosis| < 7). The within‐person attitude coefficient corresponds to the a_1_ mediation path, indexing the association between within‐person daily attitude (X) and same‐day drinking intentions (M_1_).

##### 2.3.2.2 Step 2 Paths a_2_ and d_21_: Daily Attitudes and Intentions Predicting Same‐Day Drinking

Daily alcohol consumption was simultaneously regressed on within‐person centered attitude and within‐person centered intentions. The attitude coefficient is the a_2_ mediation path, representing the effect of daily attitude (X) on same‐day drinking (M_2_) after accounting for intentions. In the same model, the intentions coefficient is the d_21_ path, representing the effect of daily intentions (M_1_) on same‐day drinking (M_2_) after accounting for attitude. To maintain a consistent within‐person metric across the full serial chain, daily drinking was operationalized as the within‐person deviation in drinks consumed; coefficients reflect the change in drinks relative to a participant's own average per one‐unit within‐person shift in the predictor and was modeled with a Gaussian distribution. Skew (1.49) and kurtosis (4.29) values for within‐person centered daily suggested an approximate normal distribution (Curran et al. 1996; |skewness| < 2, |kurtosis| < 7). Between‐person predictors were retained in this model for consistency in covariate structure across analyses, although by construction they are not expected to account for variance in a within‐person‐centered outcome.

##### 2.3.2.3 Step 3 Paths b_1_ and b_2_: Intentions and Drinking Predicting Same‐Day Consequences (Y_1_) and Blackout (Y_2_)

Each binary harm outcome was regressed on within‐person centered intentions (M_1_) and within‐person centered drinking (M_2_) simultaneously, adjusting for within‐person centered attitude (X) and all covariates. The intentions coefficient is the b_1_ path and the drinking coefficient is the b_2_ path. Both consequences and blackout were modeled with PROC GLIMMIX using a binary distribution and logit link; coefficients are on the log‐odds scale and reflect the change in the odds of the outcome per one‐unit within‐person shift in the respective predictor.

#### 2.3.3 Quantifying Specific Indirect Effects: Monte Carlo Confidence Intervals

Specific indirect effects were tested using Monte Carlo confidence intervals based on the distribution‐of‐the‐product approach (MacKinnon et al. 2004; Preacher and Selig 2012). This approach does not assume that the sampling distribution of the indirect effect is symmetric and is well suited to the product‐of‐coefficients form of mediation. Each interval was based on 10,000 simulated draws, with each path coefficient sampled from a distribution defined by its point estimate and standard error obtained from the corresponding model output. For each outcome, we computed three indirect effects: the effect of attitude on the outcome via intentions alone (a_1_ × b_1_; indirect effect 1), via drinking alone (a_2_ × b_2_; indirect effect 2), and via the full serial chain through intentions and then drinking (a_1_ × d_21_ × b_2_; indirect effect 3). An indirect effect was significant when its 95% Monte Carlo interval did not contain zero. Finally, because the harm outcomes (consequences and blackout) were modeled with logistic regression, the b_1_ and b_2_ coefficients are on the log‐odds scale, whereas the a_1_, a_2_, and d_21_ coefficients are on a linear scale; products involving these paths therefore constitute a mixed‐scale (linear × log‐odds) and should be read as indicating the direction and significance of the mediated pathway rather than a single interpretable effect‐size metric.

## 3 Results

### 3.1 Descriptive Information

Primary analyses included the 87 participants assigned to the Drinking Dashboard condition (M_age_ = 22.22, SD = 2.89; 53.4% female). Participants completed an average of 26.64 morning reports (SD = 6.23), typically ~10:00 a.m. Across the assessment period, participants drank on an average of 9.96 days (SD = 6.67) and consumed a mean of 5.27 drinks on days they drank (SD = 4.08). Twenty‐two participants reported 1+ en bloc blackout over 30 days of morning diaries (M = 1.18, SD = 0.50; range 1–3 per person), and 69 reported 1+ other alcohol‐related consequence (M = 1.39, SD = 0.60; range 1–3 per person). Specific to each consequence, hangover was the most frequently endorsed consequence (183 days), followed by embarrassment (57 days), vomiting (36 days), and passing out (25 days), injury (9 days), aggression (5 days), and emergency room visits (0 days). The sample identified as 64% White, 14% Multiracial, 3% Asian, 3% Black/African American, and 1% Middle Eastern, with 14% identifying as Hispanic/Latino. In terms of educational background, 14% had never attended college, 43% were currently enrolled, and 43% had completed a college degree. One in three participants (35%) was employed full‐time.

### 3.2 Multilevel Modeling Results

#### 3.2.1 Preliminary Analyses

Prior to conducting formal multilevel analyses, empty intercept‐only models were estimated for each of the focal variables to determine which proportion of variance was at the between‐ and within‐person levels. The ICC for attitudes toward heavy drinking was 0.30, indicating that 30% of the variance occurred between persons and the remaining 70% occurred within persons supporting the examination of attitudes as a within‐person, day‐level predictor. A similar pattern emerged for intentions (ICC = 0.19) and drinks per day (ICC = 0.19), as well as for the binary outcomes of alcohol‐related consequences (ICC = 0.23) and blackout (ICC = 0.24), with ICCs for the binary outcomes computed using the latent‐variable approach (Snijders and Bosker 2012). Across all constructs, the majority of variance resided within persons (roughly 70%–81%), supporting the use of two‐level multilevel models.

#### 3.2.2 Step 1: Daily Attitudes Predicting Same‐Day Intentions (Path a_1_)

Within‐person attitude toward heavy drinking significantly and positively predicted same‐day drinking intentions (a_1_: b = 0.748, SE = 0.019, *p* < 0.001), such that on days when a participant's attitude was more favorable than their own average, they reported stronger intentions to drink. This coefficient represents the a_1_ path. See Table 1 Path a_1_ for full results.

**TABLE 1:** Serial mediation models with daily attitude toward heavy drinking predicting consequences and blackout via daily intentions and drinking (N = 87).

		Path a_1_ Daily intentions_CWP_ (M_1_)			Path a_2_/d_21_ Daily drinking_CWP_ (M_2_)			Path b_1_/b_2_ Daily alcohol‐consequences (Y_1_)				Path b_1_/b_2_ Daily blackout (Y_2_)			
Predictor		b	SE	*p*	b	SE	*p*	b	SE	OR	*p*	b	SE	OR	*p*
	Intercept	0.108	0.098	0.272	−0.059	0.277	0.8317	**−3.718**	**0.711**	—	**< 0.001**	**−4.627**	**1.170**	—	**0.** **001**
L1	Day in study	−0.005	0.002	0.051	−0.001	0.007	0.8448	**−0.040**	**0.013**	**0.961**	**0.** **002**	0.016	0.029	1.016	0.573
	Weekend day	−0.068	0.042	0.109	0.144	0.118	0.2243	−0.265	0.208	0.767	0.203	0.353	0.452	1.423	0.435
	Daily attitudes_cwp_	**0.748**	**0.019**	**< 0.001**	**0.629**	**0.070**	**< 0.001**	−0.207	0.112	0.813	0.065	0.088	0.269	1.092	0.743
	Daily intentions_cwp_	—	—	—	**1.234**	**0.060**	**< 0.001**	**0.300**	**0.104**	**1.35**	**0.** **004**	−0.089	0.234	0.914	0.703
	Daily drinking_cwp_	—	—	—	—	—	—	**0.508**	**0.043**	**1.663**	**< 0.001**	**0.376**	**0.068**	**1.456**	**< 0.001**
L2	Sex	0.006	0.043	0.886	0.024	0.126	0.8462	**−1.067**	**0.367**	**0.344**	**0.** **004**	−0.923	0.566	0.397	0.103
	Baseline descriptive norms	0.000	0.003	0.912	−0.002	0.008	0.7613	0.015	0.021	1.015	0.478	0.015	0.027	1.015	0.574
	Mean daily attitudes_cbp_	−0.002	0.032	0.958	0.038	0.090	0.6685	**−0.586**	**0.237**	**0.557**	**0.** **014**	0.094	0.418	1.098	0.823
	Mean daily intentions_cbp_	−0.004	0.034	0.898	−0.002	0.121	0.9902	**1.714**	**0.310**	**5.55**	**< 0.001**	−0.797	0.655	0.451	0.225
	Mean daily drinking _cbp_	—	—	—	−0.012	0.043	0.7794	−0.030	0.116	0.97	0.793	−0.042	0.183	0.959	0.818

*Note:* Within‐person intentions (M_1_) and within‐person drinking (M_2_) modeled as a Gaussian deviation score. Consequences (Y_1_) and blackout (Y_2_) modeled as binary logistic outcomes (b coefficients on the log‐odds scale; OR = Odds ratio). Dashes indicate the predictor was not included in that model (the variable served as the outcome). Significant effects (*p* < 0.05) bolded.

Abbreviations: CBP, centered between‐person; CWP, centered within‐person.

#### 3.2.3 Step 2: Daily Attitudes and Intentions Predicting Same‐Day Drinking (Paths a_2_ and d_21_)

Within‐person attitude toward heavy drinking significantly predicted same‐day drinking after accounting for intentions (a_2_: b = 0.629, SE = 0.070, *p* < 0.001), and within‐person intentions significantly and positively predicted drinking (d_21_: b = 1.234, SE = 0.060, *p* < 0.001), such that on days when intentions were stronger than a participant's own average, they consumed more alcohol relative to their own typical level (See Table 1 Path a_2_/d_21_ for full results). The simple indirect effect of attitudes on drinking through intentions was significant (indirect effect = 0.924, 95% CI [0.825, 1.023]). The persistence of a significant a_2_ path after controlling for intentions indicated partial mediation of the attitude‐drinking association.

#### 3.2.4 Step 3 (Y_1_ ): Intentions and Drinking Predicting Same‐Day Consequences (Paths b_1_ and b_2_)

Within‐person intentions significantly and positively predicted the likelihood of alcohol‐related consequences (b_1_: b = 0.300, SE = 0.104, *p* = 0.004), such that stronger intentions relative to one's own average were associated with greater odds of experiencing consequences. Within‐person drinking was the strongest predictor of consequences (b_2_: b = 0.508, SE = 0.043, *p* < 0.001), such that greater same‐day consumption relative to one's own average was associated with substantially higher odds of consequences. The direct effect of within‐person attitude on consequences was not significant after controlling for intentions and drinking (b = −0.207, SE = 0.112, *p* = 0.065; See Table 1 Path b_1_/b_2_ consequences for full results). See Figure 2a for the results of each respective path from steps 1−3.

**FIGURE 2:**
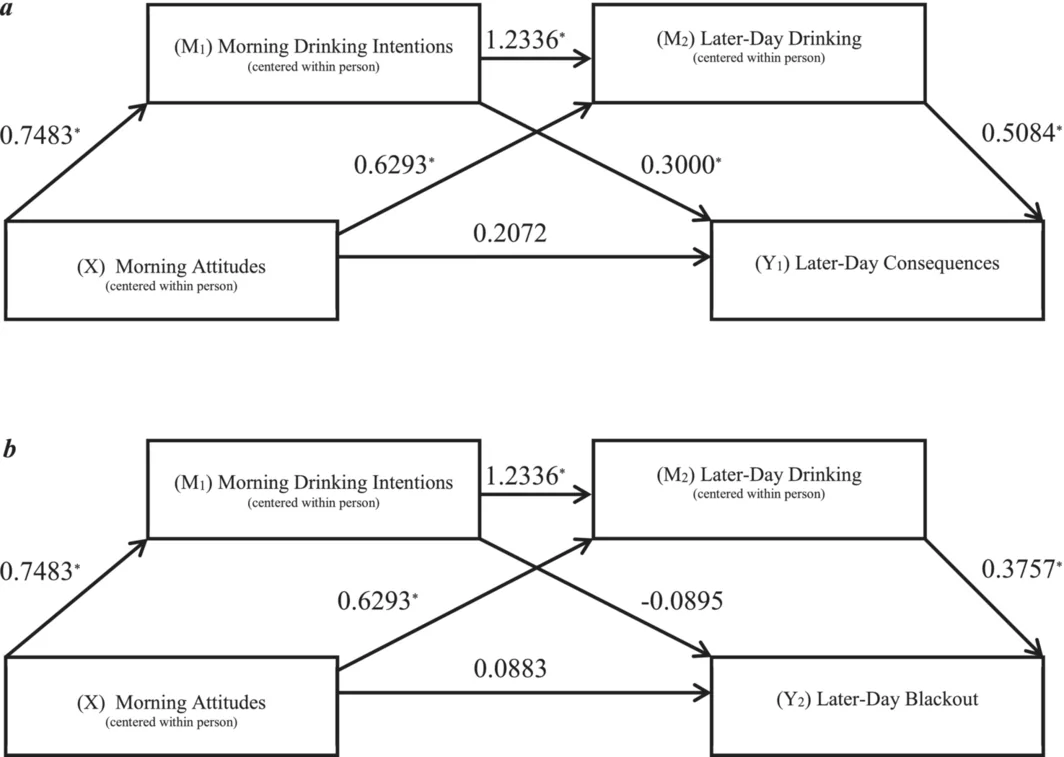
(a) Serial Mediation Model for Daily Alcohol‐consequences. *p < 0.05. All numbers are unstandardized coefficients. (b) Serial Mediation Model for Daily Blackout.

#### 3.2.5 Specific Indirect Effects: Consequences

All three specific indirect effects were significant. The indirect effect through intentions alone (a_1_ × b_1_) was significant (indirect effect = 0.224, 95% CI [0.074, 0.376]), indicating that on days when attitudes were more favorable than usual, stronger intentions contributed to greater odds of consequences. The indirect effect through drinking alone (a_2_ × b_2_) was also significant (indirect effect = 0.320, 95% CI [0.238, 0.413]). Most importantly, the focal serial indirect effect (a_1_ × d_21_ × b_2_) was significant (indirect effect = 0.469, 95% CI [0.379, 0.566]), indicating that on days when attitudes were more favorable than a participant's own average, intentions were stronger, which was associated with greater drinking, which in turn was associated with greater odds of experiencing a negative consequence.

#### 3.2.6 Sensitivity Analyses: More Common and Less Common Consequences

In order to evaluate whether aggregating and modeling consequences of differing frequency influenced our conclusions, we conducted additional sensitivity analyses wherein we separated more commonly endorsed consequences (hangover, embarrassment, vomiting) from less commonly endorsed consequences (passing out, injury, aggression, emergency‐service use), a grouping informed by prior work examining daily alcohol‐related consequences (Richards et al. 2026). For more commonly endorsed consequences, all three indirect effects were significant, closely reproducing the primary model (serial indirect effect = 0.447, 95% CI [0.359, 0.541]). For less commonly endorsed consequences, the drinking‐mediated and serial pathways remained significant despite the lower endorsement rate of that consequence grouping (serial indirect effect = 0.301, 95% CI [0.170, 0.436]); only the intentions‐only pathway fell just short of significance. These analyses indicate that the mediated associations were robust across consequences of differing frequency. Please see Table S1 for the full results of these models.

#### 3.2.7 Step 3 (Y_2_ ): Intentions and Drinking Predicting Same‐Day Blackout (Paths b_1_ and b_2_)

Within‐person intentions did not significantly predict the likelihood of blackout after controlling for drinking (b_1_: b = −0.089, SE = 0.234, *p* = 0.703). Within‐person drinking, however, significantly and positively predicted blackout (b_2_: b = 0.376, SE = 0.068, *p* < 0.001), such that greater same‐day consumption relative to one's own average was associated with substantially higher odds of blackout. The direct effect of within‐person attitude on blackout was not significant (b = 0.088, SE = 0.269, *p* = 0.743; see Table 1 Path b_1_/b_2_ blackout for full results). See Figure 2b for the results of each respective path from steps 1−3.

#### 3.2.8 Specific Indirect Effects: Blackout

The indirect effect through intentions alone (a_1_ × b_1_) was not significant (indirect effect = −0.067, 95% CI [−0.404, 0.272]). However, the indirect effect through drinking alone (a_2_ × b_2_) was significant (indirect effect = 0.237, 95% CI [0.145, 0.340]), as was the focal serial indirect effect (a_1_ × d_21_ × b_2_; indirect effect = 0.347, 95% CI [0.221, 0.476]). The significant serial pathway indicates that on days when attitudes were more favorable than usual, intentions were stronger, which was associated with greater drinking, which in turn predicted greater odds of blackout.

## 4 Discussion

The present study leveraged a 30‐day diary design to examine whether within‐person fluctuations in drinking attitudes predict same‐day alcohol‐related consequences and blackouts via a serial mediation through drinking intentions and alcohol consumption. Across all models, results were consistent with the hypothesized TPB‐informed serial chain: on days when attitudes toward drinking were more favorable than usual, drinking intentions were stronger, which predicted greater drinking, which in turn predicted greater likelihood of consequences and blackout. These findings provide day‐level support for a deliberative, intention‐based pathway from attitudes to alcohol‐related harm, consistent with the Theory of Planned Behavior framework (McEachan et al. 2011). They also extend a growing body of intensive longitudinal research demonstrating that alcohol‐related cognitions fluctuate meaningfully within individuals and represent proximal determinants of drinking behavior and alcohol‐related risk (e.g., Merrill et al. 2023; Monk and Heim 2014), while specifically building on emerging work highlighting within‐person fluctuations in drinking attitudes (DiBello et al. 2025, 2026).

A consistent and theoretically important finding across all models was that within‐person deviations from one's own attitude mean were robustly associated with same‐day intentions and with drinking even after accounting for intentions. This pattern is consistent with findings from DiBello et al. (2025), who found that within‐person fluctuations in attitude toward heavy drinking over 12 months were associated with more drinking, greater odds of blackout, and more consequences, while between‐person attitude effects were less consistent across outcomes. It also extends the findings of DiBello et al. (2026) by demonstrating that day‐level, within‐person associations between attitudes and drinking outcomes operate through a deliberative, intention‐based pathway. Together with this prior work, these findings suggest that attitudes toward drinking function as both relatively stable individual differences and dynamic evaluations that fluctuate across contexts and situations. This interpretation is theoretically consistent with accessibility models of attitudes, which posit that attitudes vary in their momentary activation depending on situational cues and contextual factors (Fazio 1990, 2007). Days characterized by unusually favorable heavy‐drinking attitudes may reflect shifts in the accessibility of positive drinking evaluations due to social opportunity, stress, or prior drinking context, translating rapidly into intentions and behavior. The null between‐person effects observed here and in prior work (DiBello et al. 2025, 2026) further suggest that stable individual differences in attitude valence, while important, are insufficient to explain day‐to‐day variation in drinking risk, underscoring the importance of within‐person (e.g., daily, momentary) assessment approaches.

Drinking intentions emerged as a robust mediator of the attitude–drinking relationship and, for consequences specifically, as a significant direct predictor of harm. The simple indirect effect of attitudes on drinking through intentions was large, suggesting that the deliberative, TPB‐informed pathway (i.e., favorable attitudes activating intentions, which drives drinking) is a powerful within‐person process at the daily level. This extends the prospective between‐person findings that attitudes toward heavy drinking predicted change in blackout frequency through intentions over time (DiBello et al. 2023), to the within‐person daily level. Taken together, these findings suggest that the attitude–intention–behavior chain identified in original conceptualizations of the TPB operates not only as a stable individual difference process but also as a dynamic, day‐to‐day mechanism.

Notably, intentions predicted consequences directly and independently of drinking in the consequences model, but did not operate similarly when examining blackout as the outcome. This differentiation is potentially theoretically meaningful. Indeed, consequences (as measured here) encompass a broad range of negative outcomes that may arise from the intentional decision to drink heavily, independent of the specific quantity consumed. Blackout, by contrast, is largely determined by physiological blood alcohol concentration, requiring sufficient consumption to impair memory consolidation regardless of intent. The observed pattern is consistent with prior work noting that blackouts are uniquely linked to the amount consumed rather than the broader context of drinking (Wetherill and Fromme 2016). This suggests that the pathways to consequences and blackout, while overlapping, are not identical. We hypothesize that experiencing consequences broadly is more sensitive to the motivational and decisional context of drinking, while blackout is more directly constrained by consumption quantity.

Although same‐day drinking was the strongest predictor of both consequences and blackout in all models, the indirect effect of attitudes on both consequences and blackout remained significant through drinking alone. This finding highlights the importance of modeling the 1‐1‐1‐1 serial mediation with drinking as an explicit mediator, rather than a control variable, as it allows the pathways to each outcome to be distinguished. Such unique pathways would be lost in a 1‐1‐1 model treating drinking as a covariate. The finding that attitudes are linked to consequences and blackout through drinking is consistent with DiBello et al. (2026), who found similarly strong within‐person associations between attitudes and drinking quantity, with attitudes associated with a 99% increase in expected drinks on days when attitudes were more favorable than usual. The present findings extend this work by establishing that these attitude‐driven increases in drinking translate into measurable increases in the probability of consequences and blackout via the drinking pathway. Furthermore, the serial indirect effect (from attitudes to intentions to drinking to outcomes) was significant for both consequences and blackout, providing evidence for the full TPB‐informed causal chain. Modeling drinking as a mediator rather than a covariate, as is standard practice in the alcohol literature, makes explicit the primary behavioral pathway through which cognitive antecedents translate into harm.

Descriptive norms assessed at baseline only were included as a between‐person covariate in all models, consistent with the TPB framework and prior work demonstrating norms as a reliable predictor of alcohol consumption among young adults (Neighbors et al. 2007). However, norms were non‐significant in all models (all ps > 0.45) and their inclusion did not alter primary effects. This null finding is consistent with DiBello et al. (2025), who found that while between‐person norms predicted drinks per week, within‐person norms showed weaker and more inconsistent associations with outcomes in some models. In the present daily diary context, baseline norms were assessed only once and therefore could not capture within‐person variation in perceived peer drinking, which may carry more proximal influence. Future research could examine whether daily fluctuations in perceived peer drinking alter the attitude–intention–drinking chain found within the current work.

### 4.1 Clinical Implications

The present findings carry several important implications for prevention and intervention. First, the strong within‐person attitude–intention–drinking chain suggests that interventions targeting days with higher positive attitudes are worth studying in this population. Consistent with the hypothesis that intervening on attitudes may be effective, our team recently found that the digital health intervention tested in this study (the Drinking Dashboard) reduced favorable attitudes toward heavy drinking, and that these attitude changes mediated subsequent reductions in high‐intensity drinking (DiBello et al. 2026). Based on results of this analysis, intentions may be the within‐person mechanism through which attitude changes reduce drinking and downstream harm. Ecological momentary interventions that detect and respond to daily attitude elevations in real time, delivering targeted content to reduce favorable appraisals of drinking in the moment, may represent a promising avenue for prevention and intervention. The finding that intentions predicted consequences independently of drinking quantity also lends support to prevention intervention strategies targeting intentions; for example, through implementation intentions or if‐then planning strategies (Gollwitzer 1999).

### 4.2 Limitations

Several limitations should be noted. First, although we can confirm that attitudes and intentions were assessed prior to later‐day drinking and consequences, attitudes and intentions were assessed at the same time point, as were drinking and consequences, within the single daily reports precluding strict temporal ordering. However, we model the attitudes ➔ intentions ➔ behavior chain consistent with the Theory of Planned Behavior (Ajzen 1991). It would be interesting to see if future work assessing these constructs at different points within a day, or immediately prior to a drinking episode, shows associations similar to those found in the current work. Second, the sample was limited to heavy‐drinking young adults with recent blackout history enrolled in the intervention arm of a randomized trial, which may limit generalizability to the broader population of drinkers. Third, all measures were self‐reported, which introduces potential recall and social desirability biases; this limitation may be particularly relevant given the elevated prevalence of blackouts in this sample, which can impair memory for drinking episodes. Fourth, we did not assess norms or perceived behavioral control at the daily level, limiting the comprehensiveness of the test of the TPB model. Fifth, it should be noted that the attitude and intentions items differed with respect to the quantity of drinking (i.e., attitudes were assessed specific to heavy drinking (4/5+ drinks), intentions were specific to any drinking). The different levels of specificity may not be optimal from a theoretical standpoint; however, we believe it would attenuate, rather than inflate, the found association. In the future, using matched drinking thresholds in day‐level assessments of these constructs would bolster the support for the associations found here. Finally, it should be noted that all data modeled in the current work come from participants who were enrolled in an intervention that reduced favorable attitudes toward heavy drinking (DiBello et al. 2025), which could have influenced the present associations. However, that concern is tempered by the within‐person analyses documented here. That is, the present models examined day‐to‐day deviations from each person's own average and adjusted for day in study. The findings thus reflect within‐person covariation between the cognitive constructs and drinking outcomes rather than mean‐level intervention effects.

## 5 Conclusion

The present study provides support for a TPB‐informed serial mediation model in which within‐person attitude fluctuations predict same‐day drinking and alcohol‐related harm through daily drinking intentions and alcohol consumption. Together with prior work, these findings suggest that attitudes toward heavy drinking should be thought of as operating at multiple levels. Stable between‐person differences remain important for understanding who is at greater risk, whereas within‐person fluctuations may help explain when individuals are most likely to engage in risky drinking and experience alcohol‐related harm. Additionally, consistent with the TPB, intentions serve as the key deliberative mechanism at the day level. Together, these findings advance a comprehensive understanding of the within‐person cognitive pathway to alcohol‐related harm and highlight daily attitudes and intentions as promising targets for real‐time, ecologically sensitive intervention efforts.

## Funding

This work was supported in part by the National Institute on Alcohol Abuse and Alcoholism (grant numbers R34 AA028077‐01).

## Conflicts of Interest

The authors declare no conflicts of interest.

## Supporting information


**Table S1:** Serial mediation models with daily attitude toward heavy drinking predicting more common consequences and less common consequences via daily intentions and drinking.

## Data Availability

The data that support the findings of this study are available from the corresponding author upon reasonable request.
