# Supplementary material for: From Daily Attitudes to Alcohol‐Related Harm: A Within‐Person Serial Mediation Test Informed by the Theory of Planned Behavior
Source: Alcohol Clin Exp Res (Hoboken). 2026 Oct 27;50(11):e70431. doi: 10.1111/acer.70431 (PMC13714157; doi:10.1111/acer.70431)
Supplement: Supplementary file 1 [file ACER-50-0-s001.docx]

***Table S1.*** Serial mediation models with daily attitude toward heavy drinking predicting more common consequences and less common consequences via daily intentions and drinking

|  |  | **Path a_1_**  Daily Intentions_CWP_ (M_1_) | | | **Path a_2_ / d_21_**  Daily Drinking_CWP_ (M_2_) | | | **Path b_1_ / b_2_**  More Common Consequences (Y_1_) | | | | **Path b_1_ / b_2_**   Less Common Consequences (Y_2_) | | | |
| --- | --- | --- | --- | --- | --- | --- | --- | --- | --- | --- | --- | --- | --- | --- | --- |
|  | **Predictor** | ***b*** | **SE** | ***p*** | ***b*** | **SE** | ***p*** | ***b*** | **SE** | **OR** | ***p*** | ***b*** | **SE** | **OR** | ***p*** |
|  | Intercept | 0.108 | 0.098 | .272 | -0.059 | 0.277 | .8317 | -3.364 | 0.684 | — | <.001 | -7.038 | 1.497 | — | <.001 |
| L1 | Day in Study | -0.005 | 0.002 | .051 | -0.001 | 0.007 | .8448 | **-0.036** | **0.013** | **0.964** | **.005** | **-0.065** | **0.030** | **0.937** | **.031** |
|  | Weekend Day | -0.068 | 0.042 | .109 | 0.144 | 0.118 | .2243 | -0.284 | 0.209 | 0.753 | .175 | 0.151 | 0.438 | 1.163 | .730 |
|  | Daily Attitudes_cwp_ | **0.748** | **0.019** | **<.001** | **0.629** | **0.070** | **<.001** | -0.179 | 0.112 | 0.836 | .111 | -0.210 | 0.255 | 0.811 | .412 |
|  | Daily Intentions_cwp_ | — | — | — | **1.234** | **0.060** | **<.001** | **0.262** | **0.105** | **1.299** | **.012** | 0.442 | 0.239 | 1.556 | .064 |
|  | Daily Drinking_cwp_ | — | — | — | — | — | — | **0.484** | **0.043** | **1.623** | **<.001** | **0.326** | **0.071** | **1.385** | **<.001** |
| L2 | Sex | 0.006 | 0.043 | .886 | 0.024 | 0.126 | .8462 | **-0.910** | **0.351** | **0.402** | **.010** | 0.729 | 0.383 | .188 | 0.729 |
|  | Baseline Descriptive Norms | 0.000 | 0.003 | .912 | -0.002 | 0.008 | .7613 | 0.010 | 0.020 | 1.010 | .632 | **0.039** | **1.025** | **.518** | **0.039** |
|  | Mean Daily Attitudes_cbp_ | -0.002 | 0.032 | .958 | 0.038 | 0.090 | .6685 | **-0.654** | **0.229** | **0.520** | **.004** | 0.449 | 0.789 | .597 | 0.449 |
|  | Mean Daily Intentions_cbp_ | -0.004 | 0.034 | .898 | -0.002 | 0.121 | .9902 | **1.571** | **0.295** | **4.809** | **<.001** | 0.576 | 4.254 | .012 | 0.576 |
|  | Mean Daily Drinking _cbp_ | — | — | — | -0.012 | 0.043 | .7794 | -0.032 | 0.112 | 0.969 | .777 | 0.209 | 0.992 | .971 | 0.209 |

***Note.*** *CWP = centered within-person; CBP = centered between-person. Within-person intentions (M_1_) and within-person drinking (M_2_) modeled as a Gaussian deviation score. More Common Consequences (Y_1_) and Less Common Consequences (Y_2_) modeled as binary logistic outcomes (b coefficients on the log-odds scale; OR = Odds ratio). Dashes indicate the predictor was not included in that model (the variable served as the outcome). Significant effects (p < .05) bolded.*
